# Does hand position affect orienting when no action is required? An electrophysiological study

**DOI:** 10.3389/fnins.2022.982005

**Published:** 2023-01-06

**Authors:** Catherine L. Reed, John P. Garza, William S. Bush, Natasha Parikh, Niti Nagar, Shaun P. Vecera

**Affiliations:** ^1^Department of Psychological Science, Claremont McKenna College, Claremont, CA, United States; ^2^BUILDing SCHOLARS Center, The University of Texas, El Paso, TX, United States; ^3^Department of Psychological and Brain Sciences, The University of Iowa, Iowa City, IA, United States

**Keywords:** attention, embodied attention, orienting, haptic, encephalography (EEG), event-related potential (ERP)

## Abstract

Previous research has shown that attention can be biased to targets appearing near the hand that require action responses, arguing that attention to the hand facilitates upcoming action. It is unclear whether attention orients to non-targets near the hand not requiring responses. Using electroencephalography/event-related potentials (EEG/ERP), this study investigated whether hand position affected visual orienting to non-targets under conditions that manipulated the distribution of attention. We modified an attention paradigm in which stimuli were presented briefly and rapidly on either side of fixation; participants responded to infrequent targets (15%) but not standard non-targets and either a hand or a block was placed next to one stimulus location. In Experiment 1, attention was distributed across left and right stimulus locations to determine whether P1 or N1 ERP amplitudes to non-target standards were differentially influenced by hand location. In Experiment 2, attention was narrowed to only one stimulus location to determine whether attentional focus affected orienting to non-target locations near the hand. When attention was distributed across both stimulus locations, the hand increased overall N1 amplitudes relative to the block but not selectively to stimuli appearing near the hand. However, when attention was focused on one location, amplitudes were affected by the location of attentional focus and the stimulus, but not by hand or block location. Thus, hand position appears to contribute only a non-location-specific input to standards during visual orienting, but only in cases when attention is distributed across stimulus locations.

## 1. Introduction

Spatial attention is crucial for everyday behavior. It plays an important role in perception by focusing processing toward the most important objects at a given moment. However, the relationship between your body and objects in the environment, or body position, is also important for interacting with those objects selected by attention. Hand position can affect visual processing in at least two ways, via multisensory integration early in processing potentially through bimodal neurons (Graziano and Gross, 1998) or through more top-down allocations of attentional resources across the visual field (Mangun et al., 1993). Previous research has shown that attention can be biased to targets appearing near the hand that require action responses and has argued that attention to locations near the hand facilitates upcoming action (Reed et al., 2013, 2018; Vyas et al., 2019). It is unclear whether attention orients to non-targets near the hand not requiring responses. Using electroencephalography/event-related potentials (EEG/ERP), this study investigated whether hand position affected visual orienting to non-targets under conditions that manipulated the distribution of attention (Simon-Dack et al., 2009; Qian et al., 2012; Reed et al., 2013, 2018; Vyas et al., 2019).

How might hand position control attention? Attentional control is a longstanding issue within the attentional literature, in which attention can be directed to stimuli based on either stimulus factors (exogenous factors) or goal relevance (endogenous factors or ‘attentional set’). Although most theorists agree that both stimulus-driven factors and attentional set control attention, the relative contributions of these control parameters are hotly debated (Awh et al., 2012; Vecera et al., 2014; Luck et al., 2021). Some argue that stimulus-driven control is automatic and occurs independently of attentional set (e.g., Theeuwes, 1994). For example, attentional set effects emerge only after the initial bottom-up capture. Others argue that attentional capture is under the control of the participants’ goals (e.g., Folk et al., 1992; Leber and Egeth, 2006) and that stimulus-driven capture occurs only for stimuli that match an attentional set. More recently, researchers have examined how potential action can contribute to higher-level attentional control (Hanning et al., 2022). Against this theoretical framework, it becomes important to know how body position fits into the traditional attentional control scheme. Does the hand act as a control parameter separate from stimulus-driven and goal-directed parameters? If so, might hand position override capture by stimulus factors and might it capture attention independent of attentional set?

The possibility that hand position could affect attention was initially suggested by electrophysiological recordings from non-human primates that identified populations of neurons that respond to both tactile and visual stimuli, located in cortical regions supporting a multimodal system for upcoming action: parietal cortex, premotor cortex, and the putamen (Graziano and Gross, 1994; Fogassi et al., 1996; Duhamel et al., 1998; Graziano, 1999; Graziano and Cooke, 2006). In macaques, bimodal visuotactile neurons are distinguished by their characteristic response properties in peripersonal space (Rizzolatti et al., 1981; Gentilucci et al., 1988; Fogassi et al., 1992, 1996; Graziano and Gross, 1993, 1994, 1998; Iriki et al., 1996, 2001; Obayashi et al., 2000). These neurons not only respond to tactile stimulation on the hand, but also to visual stimuli appearing on or near the hand (Graziano and Gross, 1993, 1994). They also have spatially graded responses in that the size of the response decreases as the visual stimulus appears progressively further from the hand. Further, these responses are specific to the hand and do not occur when the subject’s arm is located away from the target, or when an artificial arm is placed near the visual target (Graziano et al., 2000).

Attention may be directed by the hand because bimodal neurons encode space based on hand-centered coordinate systems. Visual stimuli appearing near the hand may elicit activation from bimodal neurons that respond specifically to regions on and near the hand, from visual neurons representing visual inputs, and from proprioceptive neurons representing limb position. Thus, visual stimuli appearing in space near the hand may produce a stronger overall neural response than visual targets appearing far from the hand that do not engage bimodal neurons.

In humans, behavioral, psychophysical, and EEG studies have shown that body and hand position affect attention and visual processing to targets in nearby space (di Pellegrino and Frassinetti, 2000; Grubb and Reed, 2002; Reed et al., 2006, 2010; Abrams et al., 2008; Grubb et al., 2008; Cosman and Vecera, 2010; Brockmole et al., 2013; Garza et al., 2013; Bush and Vecera, 2014; Stettler and Thomas, 2017; Garza et al., 2018). Reed et al. (2006) demonstrated the hand proximity effect, – the facilitation or bias of processing for targets near the hand in a purely visual, predictive covert-orienting task. Participants held one hand next to one of two lateralized target locations. Hand location changed attention to the space near the hand. Responses were faster for targets appearing next to the palm in graspable space compared to targets appearing far from the palm outside of graspable space. Attention advantages were attenuated when the hand moved away from the visual stimulus, proprioceptive cues to hand position were eliminated but visual inputs to hand location remained, or hand location was not visible. These behavioral findings were consistent with the properties of visuotactile bimodal neurons: hand centered (decreasing strength as the target moved away from the hand) and multimodal (sensitive to both visual and haptic/proprioceptive information).

Subsequent research confirmed hand proximity influences other attentional and cognitive processes associated with action when participants respond to target stimuli. Visual stimuli appearing near the hand alters the perception of objects near the hand, allowing these objects to appear more figure like (Cosman and Vecera, 2010). Stimuli near the hand elicit attentional shifting (Lloyd et al., 2010), attentional prioritization of space (Abrams et al., 2008), and more accurate detection and discrimination of visual stimuli (Dufour and Touzalin, 2008). Action-relevant properties of the hand or tools appear to be important for the hand proximity effect (e.g., Reed et al., 2010; Thomas, 2015; Vyas et al., 2019). Finally, the hand proximity effect is also affected by attentional set, as manipulated by task instructions (Garza et al., 2013). These findings are consistent with the finding that functional actions associated with an object and the action intentions of the observer influence early and low-level visual and attentional processes in the brain (Bortoletto et al., 2011; Goslin et al., 2012; Gozli et al., 2012).

Early perceptual and later cognitive influences of the hand proximity effect on attention have also documented in studies examining ERP components related to sustained attention and object processing (P1, N1, and P3). The P1, a positive deflection occurring approximately 100 ms after stimulus onset is associated with visual stimulus detection. The N1, a negative deflection occurring between 140 and 200 ms in occipitoparietal electrodes, is associated with visuo-tactile or visual-proprioceptive integration, visual attention and stimulus discrimination processes during early sensory processing in the visual and parietal cortices (Eimer, 1994; Kennett et al., 2001; Simon-Dack et al., 2009). The P3, a positive inflection occurring between 300-500 ms in parietal electrodes, is associated with event categorization and higher-order cognitive influences such as task relevance, attentional distributions and motivation (Kok, 2001). Hand location near a visually presented stimulus increases the amplitude of early contralateral components (P1, N1; Reed et al., 2013) without discriminating between target (i.e., stimuli that require action) and non-target (i.e., stimuli that do not require action) stimuli and also increases the amplitude of P3 amplitudes for targets relative to non-target stimuli (Reed et al., 2013, 2018; Vyas et al., 2019).

**FIGURE 1:**
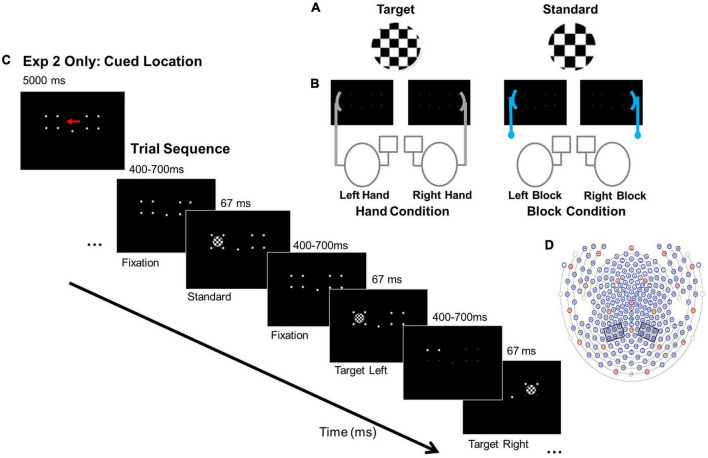
Experimental conditions, paradigm, and electrode clusters. **(A)** Example stimuli. **(B)** Anchor conditions: Hand and Block. For example, in the “Right Hand” Hand condition the right hand is near one target location and responses are made with the left index finger. In the “Right Block” Block condition, the block is placed near the right target location and responses are made with the left index finger. **(C)** Paradigms for Experiments 1 and 2: Experiment 2 cue for focal attention to the left location and basic trial sequence. In Experiment 1 participants detected targets in left and right locations. **(D)** 256- channel locations for EGI Hydrocel Geodesic EEG System 300 (Electrical Geodesic Inc., Eugene, OR). Left and right hemisphere electrode clusters are indicated by the gray boxes.

Here, we examine whether hand position affects visual orienting under distributed and focused attention conditions for stimuli that do not require actions. Also, measuring evoked responses in the absence of any response eliminates any late, post-perceptual biases. We use a variant of an attentional paradigm in which participants were instructed to monitor locations for infrequently occurring targets while non-target standards also appeared while EEG was collected (see Hillyard et al., 1973; also see Luck et al., 2000). Typical results from this paradigm were reported by Mangun et al. (1993), who manipulated the distribution of attention by asking participants to either equally divide their attention across two stimulus locations or to only one location. Participants exhibited larger P1 and N1 amplitudes when the non-targets appeared at an attended compared to an unattended location, indicating that attention enhanced stimulus perception. To focus on interactions between hand location and visual attention, we added a visual anchor manipulation (Figure 1A: one hand or block was placed on one side of a visual display while participants monitored the either the entire display (Experiment 1) or one side of the display (Experiment 2) (Figure 1B). If hand position increased the amplitudes of the P1 and N1 components relative to block position, this would suggest that hand position influences perception.

In Experiment 1 participants spread attention across two display locations for infrequently occurring targets with either a hand or a block was placed next to one display location. If hand location affected visual orienting to the hand without a functional component (i.e., ERPs to standards), we predicted that hand location, but not block location, would prioritize ERP amplitudes for standards overall, and especially for standards appearing near the hand. Experiment 2 removed the “search” component and cued participants to attend to one display location (left or right); hand and block position was orthogonal to the attended location. If hand position can compete with attentional set, then P1 and N1 amplitudes should depend on both hand position and set producing larger amplitudes for standards appearing near the hand. However, it is also possible that hand position could have stronger effects on visual orienting when visual attention is more distributed across locations as there would be more reason to integrate haptic with visual inputs.

## 2. Materials and methods

### 2.1. Participants

In Experiment 1, 22 right-handed volunteers (16 female, μ_*age*_ = 20.0 years, *SD* = 1.41). In Experiment 2, 17 right-handed volunteers (7 female, μ_*age*_ = 20.25 years, *SD* = 2.71) participated for partial course credit. All reported normal or corrected-to-normal vision and no previous head trauma. This study was approved by institutional review boards at Scripps College, Claremont McKenna College, and University of Iowa. To ensure task compliance, four participants were excluded from Experiment 1 because they had lower than 70% accuracy; no Experiment 2 participants were excluded. In addition, one participant from Experiment 1 and three participants from Experiment 2 were excluded because of excessive data artifacts. Thus, 17 data sets from Experiment 1 and 14 data sets from Experiment 2 were submitted to analysis.

### 2.2. Stimuli and apparatus

Stimuli were presented in a fully lit room on a 27 cm × 34 cm monitor (43.5 cm diagonal) using E-Prime 2.0 software (Psychology Software Tools Inc., PA) and the Pstnet SRbox to record responses. Stimuli were white, circular checkerboards, 5.72° visual angle in diameter (Figure 1A). Standard stimuli had checks 0.08° x 0.08° visual angle; target stimuli were identical to the standard stimuli but had checks 0.11° x 0.11° visual angle. Standards comprised 85% of trials; targets comprised 15% of trials. Stimuli were presented against a black background, their outer edge either 13.7° to the left or right of a central fixation dot. They were presented in pseudo-random order with the constraint that targets could not appear more than three times consecutively.

The non-hand, visual anchor (i.e., block condition) was a V-shaped wooden block constructed of two 9 cm x 1.7 cm pieces of balsa wood configured in an obtuse, 150° angle to match the general shape and size of a relaxed hand (∼18 cm in overall length). It was positioned next to the computer monitor, lateral to stimulus locations, on a ring stand (Figure 1B).

### 2.3. Procedure

Participants were seated 40 cm away from the monitor with body midlines aligned with the center of the monitor. In both experiments, they performed a modified go-no go visual attention paradigm with a hand or block anchor manipulation (Mangun et al., 1993; Reed et al., 2006) while EEG data were collected. Participants maintained focus on a central fixation dot. Stimuli appeared to the left or right of fixation for 67 ms, with intertrial intervals (ITIs) varying randomly between 400 - 700 ms. In Experiment 1, visual attention was distributed across two stimulus locations. Participants monitored the entire display and pressed a response key with their index finger when they detected an infrequently occurring target. In Experiment 2, participants performed the same paradigm with visual attention focused on either the right or left location and only detected targets in the focal location. An arrow cue, presented for 5,000 ms, preceded each block of trials to indicate whether to focus on either the left or the right location (Figure 1C). Participants monitored the cued side of the display and pressed a response key with their index finger only when they detected a target.

To address potential contributions of tactile and proprioceptive information to the attention orienting response, either a hand or a block anchor was placed adjacent to the right or left stimulus location (Figure 1B). For hand conditions, participants placed a hand against the screen next to the right or left stimulus location in a relaxed “grasp” with the palm facing the stimuli location and elbow resting on a pad. For block conditions, a block was placed in the same right or left screen location.

Participants received one block of practice for the right “hand” condition and one for the right “block” condition. Experimental trials followed with 16 blocks of 52 trials (7 targets, 45 non-targets). Blocks were short to avoid fatigue. Participants could rest between blocks. Each condition was presented four times for a total of 16 blocks (832 stimuli: 112 targets + 720 standards). To avoid habituation, blocks were presented in pseudorandom order so that the same hand/block condition could not be presented consecutively. Experiment 1 had 16 conditions: anchor (2: hand, block) x anchor side (2: left, right) x stimulus (2: target, non-target) x stimulus side (2: left, right). Experiment 2 had 32 conditions: cue side (2: right, left) x anchor (2: hand, block) x anchor side (2: left, right) x stimulus (2: target, non-target) x stimulus side (2: left, right).

### 2.4. EEG recording and data processing

EEG data were acquired using a high-impedance EGI 256-channel Hydrocel Geodesic EEG System 300 (Electrical Geodesic Inc., Eugene, OR). The EOG was recorded from electrodes located above and below each eye. The EEG sampling frequency was 250 Hz with a hardware bandpass filter from 0.1 Hz to 100 Hz. Impedances were kept below 80 Hz. EEG and EOG data were processed offline using NetStation 4.4.2 (Electrical Geodesic Inc., Eugene, OR). Continuous data were filtered with a 40-Hz low-pass filter and segmented from 200 ms pre-stimulus onset to 900 ms following stimulus onset with an offset of 19 ms.

Data were analyzed offline using the EEGLAB toolbox (Delorme and Makeig, 2004) and ERPLAB toolbox^1^. Bad channels were identified and interpolated using the spherical spline method. No participant had bad channels in the electrode clusters of interest. Independent components analysis (ICA SOBI) was used to correct for eye blink, eye movement, muscle noise, and electrical noise artifacts. Visual inspection confirmed artifact removal. Remaining trials were baseline corrected and re- referenced to the average of all electrode sites.

## 3. Results

### 3.1. Behavioral data

Trials in which response times were less than 150 ms and longer than 800 ms were attributed to anticipatory or inattention responses respectively, and excluded from analysis (less than 1% of data).

Examination of accuracy data for Experiments 1 and 2 confirmed that focused attention (Experiment 2: block = 92%, hand = 93%) produced more accurate target detection than distributed attention (Experiment 1: block = 83%, hand = 84%) with no differences between hand and block conditions.

### 3.2. ERP data

Given the small number of target trials and potential motor artifacts from the responses, only non-target data (no response trials) from correct trials were analyzed. Average waveforms were calculated for each participant and condition. Previous studies (Simon-Dack et al., 2009; Reed et al., 2013; Vyas et al., 2019) and visual analysis of the grand average waveforms were used to select the left hemisphere (LH) and right hemisphere (RH) electrode clusters that correspond with parieto-occipita bimodal neuron regions (Simon-Dack et al., 2009). Electrodes 140, 141, 151, 152, 160, and 161 formed the RH cluster; 97, 98, 99, 107, 108, and 109 formed the LH cluster (Figure 1D). These electrodes correspond roughly to PO3, PO4, PO7, and PO8 international 10-10 system (Luu and Ferree, 2005) and are consistent with other visual attention (Eimer, 1994), and visuotactile multisensory integration studies (Kennett et al., 2001; Simon-Dack et al., 2009; Blini et al., 2021).

We calculated mean amplitudes for the P1 (90 ms-130 ms) and N1 (140 ms-185 ms). Early influences of the hand for targets occur consistently at the N1 in contralateral hemispheres (Reed et al., 2013, 2017, 2018; Vyas et al., 2019). To focus on our hypotheses regarding differential processing near the hand and the findings from our previous studies (Graziano and Gross, 1998; Reed et al., 2013, 2018; Vyas et al., 2019), we collapsed over anchor side and stimulus side for amplitudes in the contralateral hemisphere. We calculated an anchor proximity factor where near proximity indicates that the anchor and the stimulus were located on the same side and far proximity indicates that the anchor is on the opposite side of the stimulus location. We report the full anchor type (2: Hand, Block), anchor side (2: left, right), stimulus side (2: left, right) and hemisphere (2: LH, RH) analyses in the Supplemental material.

### 3.3. Experiment 1 distributed attention

**FIGURE 2:**
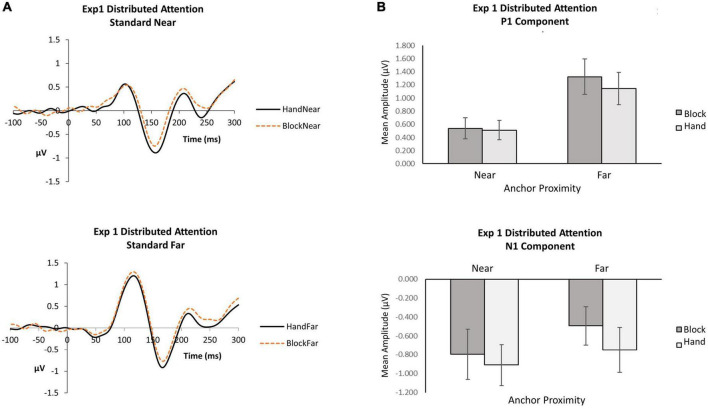
Experiment 1 distributed attention. **(A)** Grand average waveforms for hand and block conditions in near and far locations. **(B)** P1 and N1 mean amplitudes by anchor type and anchor proximity.

For P1 and N1 components, we conducted repeated measures analyses of variance (ANOVA) with anchor type (2: Hand, Block) and anchor proximity (2: near, far) using mean amplitude data (Figure 2).

*P1*. When attention was distributed, the anchor proximity effect was significant [*F*(1, 16) = 15.70, p = 0.001, 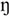
_*p*_^2^ = 0.50], with far standards (*M* = 0.1.23, *SE* = 0.25) eliciting greater P1 amplitudes than near (*M* = 0.52, *SE* = 0.15). Although the block condition (*M* = 0.93, *SE* = 0.20) produced slightly greater P1 amplitudes than the hand condition (*M* = 0.83, *SE* = 0.20), the anchor type effect did not reach significance [*F*(1, 16) = 3.84, *p* = 0.07, 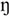
_*p*_^2^ = 0.19]. The interaction was not significant [*F*(1,16) = 1.76, *p* = 0.20, 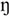
_*p*_^2^ = 0.10], suggesting that stimuli appearing near the hand did not differentially influence early visual detection processing. The data indicate that visual inputs affect the P1 more than proprioceptive inputs from the hand at the P1.

*N1*. When attention was distributed, the anchor type effect was significant [*F*(1, 16) = 9.56, *p* = 0.01, 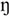
_*p*_^2^ = 0.37]: hand conditions (*M* = −0.83, *SE* = 0.21) elicited greater N1 amplitudes than block conditions (*M* = −0.65, *SE* = 0.20). A hand anchor appears to increase N1 amplitudes for both near and far stimuli compared to a block anchor. Although the near condition (*M* = −0.85, *SE* = 0.24) produced slightly greater N1 amplitudes than the far condition (*M* = −0.62, *SE* = 0.22), the anchor proximity effect did not reach significance [*F*(1, 16) = 1.27, *p* = 0.28, 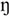
_*p*_^2^ = 0.07]. The interaction was also not significant [*F*(1,16) = 1.02, *p* = 0.33, 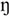
_*p*_^2^ = 0.06], suggesting that close proximity to the hand did not differentially influence the N1 for orienting to standard stimuli.

### 3.4. Experiment 2 focused attention

**FIGURE 3:**
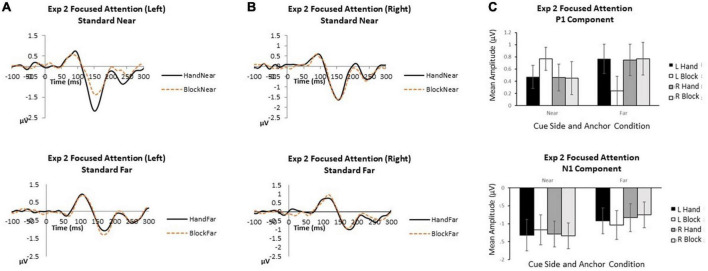
Experiment 2 focused attention. **(A)** Grand average waveforms for focused attention to the left in near and far locations. **(B)** Grand average waveforms for focused attention to the right in near and far locations. **(C)** P1 and N1 mean amplitudes by cue side, anchor type and anchor proximity. Error bars represent standard error.

For P1 and N1 components, we conducted repeated measures ANOVA with focus side (2: left, right), anchor type (2: hand, block), and anchor proximity (2: near, far) using mean amplitude data (Figure 3).

*P1*. When attention was focused on one stimulus side, no main effects or interactions reached significance (all *p*’s > 0.07).

*N1*. When attention was focused on one stimulus location, attentional focus and visual stimulus inputs overrode hand anchor effects. Anchor proximity was the only significant effect indicating that stimuli appearing near the anchor received greater attentional processing [*F*(1,13) = 4.30, *p* = 0.009, 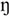
_*p*_^2^ = 0.42]. Cue side [*F*(1,13) = 0.77, *p* = 0.40, 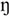
_*p*_^2^ = 0.06] was not significant, suggesting similar effects of attentional focus on left and right sides. Also, anchor type [*F*(1,13) = 0.13, *p* = 0.73, 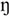
_*p*_^2^ = 0.01] was not significant, with no N1 amplitude differences between hand and block anchors. The interactions of cue side x anchor type [*F*(1,13) = 0.002, *p* = 0.96, 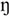
_*p*_^2^ < 0.001], cue side x anchor proximity [*F*(1,13) = 0.87, *p* = 0.37, 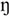
_*p*_^2^ = 0.06], anchor type x anchor proximity [*F*(1,13) = 0.40, *p* = 0.54, 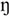
_*p*_^2^ = 0.3], and cue side x anchor type x anchor proximity [*F*(1,13) = 1.59, *p* = 0.23, _*p*_^2^ = 0.11] were not significant.

## 4. Discussion

Human perceptual and attentional systems to help us perform functional and adaptive actions in the world. Our ability to perceive, process and act on visual information in a dynamically changing environment depends on the interaction between the location of the viewed object and its proximity to our hand. Attentional processing of objects near the hand may be attributed, in part, to the combined contributions from visual, proprioceptive, tactile, and bimodal neuron found in cortical and subcortical regions of the brain that form a multimodal neural network to coordinate visual and tactile-motor systems when action is required (Fadiga et al., 2000). However, it is unknown whether hand placement itself can direct visual orienting in the absence of functional action. In this EEG/ERP study we examined whether hand position influences attentional orienting and control to stimuli and locations when responses are not required. Does the hand act as a control parameter separate from stimulus-driven and goal-directed parameters? If so, might hand position override capture by stimulus factors and might it capture attention independent of attentional set?

Using EEG/ERP, we employed a modified visual orienting paradigm in which stimuli appeared briefly and rapidly on either side of fixation while a single hand or a block was placed next to one of the two locations. We manipulated attentional set by instructing participants to focus attention across both locations (Experiment 1) or just one of them (Experiment 2). We found that hand position did not differentially affect P1 or N1 amplitudes to standards appearing near the hand regardless of whether attention was distributed or focused. In other words, there was no proximity effect of hand position relative to stimulus location. Hand position only produced a general increase in N1 amplitudes when attention was distributed across the two display locations. This general effect of the hand may have occurred because both locations were within grasping space. However, when attention was focused to a single location, visual inputs dominated processing. These findings are consistent with findings that show a narrow focus of attention restricts capture by a salient non-target (e.g., Belopolsky et al., 2007; Belopolsky and Theeuwes, 2010).

These results are consistent with a biased competition model of attention in which selective visual attention is an emergent property of competitive interactions that work in parallel across visual space. (Desimone and Duncan, 1995). Objects compete for limited processing resources and control of behavior which can be biased by both bottom-up and top-down inputs. Visual inputs regarding stimulus location dominated weaker haptic and proprioceptive inputs. Attentional set indicates whether resources should be focused on one location or spread over two. When attentional resources are spread across multiple locations, inputs from additional neural populations including vision, proprioception, touch, and bimodal neurons are associated with more bottom-up biases for resolving competition. However, when attention is focused on one location, stronger visual inputs dominate processing. An attentional set for location may provide a top-down signal that outweighs any influence from hand position.

It is also important to note that in previous studies, behavioral and neural hand proximity effects occurred for processing targets, or objects, requiring action. In these cases, the objects and locations where objects appeared were relevant for upcoming performance. In this study we focused on the orienting mechanisms of attention to non-target objects that do not require a response. The inputs from bimodal neurons, proprioception, and touch that comprise an action system are less relevant for this mechanism *per se*. It appears that it is the attentional set for orienting and the importance of upcoming action that makes the non-visual inputs from hand location relevant to processing.

## Data availability statement

The raw data supporting the conclusions of this article will be made available by the authors.

## Ethics statement

The studies involving human participants were reviewed and approved by University of Iowa Claremont McKenna College and Scripps College. The patients/participants provided their written informed consent to participate in this study.

## Author contributions

CR, JG, WB, and SV contributed equally to the conception and design of the experiment. CR and SV contributed to the writing of the manuscript. CR, NP, and NN contributed to the data collection and data analysis. All authors contributed to the article and approved the submitted version.

## Footnotes

1
http://www.erpinfo.org/erplab
