# Supplementary material for: Does hand position affect orienting when no action is required? An electrophysiological study
Source: Front Neurosci. 2023 Jan 6;16:982005. doi: 10.3389/fnins.2022.982005 (PMC9853295; doi:10.3389/fnins.2022.982005)
Supplement: Supplementary file 1 [file Data_Sheet_1.PDF]

Supplementary Material

1 Supplementary Data

Experiment 1 Distributed Attention P1 ANOVA: The anchor type (2: hand, block), anchor side (2: left, right), stimulus side (2: left, right) and hemisphere (2: LH, RH) ANOVA for P1 mean amplitude data shows significant anchor type [F(1,16)= 6.36, p = .02, η_p^2= 0.28] indicating a greater P1 amplitudes for the block over hand conditions. The stimulus side effect [F(1,16)= 15.43, p = .001, η_p^2= 0.49] shows stronger P1 amplitudes for stimuli appearing on the left side. The stimulus side x hemisphere interaction indicates larger amplitudes for left side stimuli processed in the RH [F(1,16)= 13.41, p = .002, η_p^2= 0.46]. The anchor type x anchor side x stimulus side interaction was not significant, nor were any other main effects and interactions (all p's > .18).

Experiment 1 Distributed Attention N1 ANOVA: The anchor type (2: hand, block), anchor side (2: left, right), stimulus side (2: left, right) and hemisphere (2: LH, RH) ANOVA for N1 mean amplitude data shows a significant anchor type main effect, with hand conditions producing greater N1 amplitudes than block conditions [F(1,16)= 20.36, p < .001, η_p^2= 0.56]. No other main effects and interactions approached significance (all p's > .10).

Experiment 2 Focal Attention P1 ANOVA: The cue side (2: left, right), anchor type (2: hand, block), anchor side (2: left, right), stimulus side (2: left, right) and hemisphere (2: LH, RH) ANOVA for P1 mean amplitude data indicates little influence of hand proximity effects. The anchor side effect indicated greater P1 amplitudes for right over left side anchors [F(1,13)=7.75, p = .02, η_p^2= 0.37], but the other main effects of cue side, anchor type, stimulus side and hemisphere were not significant [all F's < 1, all p's > .65]. The stimulus side x hemisphere interaction approached significance [F(1,13)= 4.20, p = .06, η_p^2= 0.24], suggesting larger contralateral amplitudes. The marginal cue side x anchor type x anchor side x stimulus side interaction suggested that attentional focus on one side with an anchor increased P1 amplitudes for visual stimuli appearing on that side with varying hand and block influences [F(1,13)= 4.23, p = .06, η_p^2= 0.25]. However, all other interactions were not significant [all F's < 3.86, p's > .07].

Experiment 2 Focal Attention N1 ANOVA: The cue side (2: left, right), anchor type (2: hand, block), anchor side (2: left, right), stimulus side (2: left, right) and hemisphere (2: LH, RH) ANOVA for N1 mean amplitude data indicates little influence of hand proximity effects. When attention was focused on one stimulus location, the cue side and the stimulus side overrode most hand contributions to any anchor effects. A significant cue x stimulus side interaction indicated increased amplitudes when the stimulus appeared on the same side as the attentional focus [F (1, 13) = 5.98, p = .03, η_p^2 = 0.32]. A significant anchor type x stimulus side interaction indicated larger N1 amplitudes for the hand condition when the stimulus appeared on the left side [F (1, 13) = 5.98, p = .03, η_p^2 = 0.32]. The cue x hemisphere interaction [F (1, 13) = 4.49, p = .05, η_p^2 = 0.26] indicated generally larger amplitudes for electrodes contralateral to the attentional cue and that participants maintained the instructed attentional set. Similarly, a significant stimulus side x hemisphere interaction [F (1, 13) = 8.22, p = .01, η_p^2 = 0.39] showed increased amplitudes in the cluster contralateral to the visual stimulus. An anchor type x stimulus side x hemisphere interaction [F (1, 13) = 7.56, p = .01, η_p^2 = 0.37] shows that although the hand condition produces equivalent N1 amplitudes for stimuli on both sides, the block

condition produces larger amplitudes for stimuli on the right. No other main effects nor interactions reached significance [all p's > .07].
